# A practical approach to remote ischemic preconditioning and ischemic preconditioning against myocardial ischemia/reperfusion injury

**DOI:** 10.14440/jbm.2016.149

**Published:** 2016-10-31

**Authors:** Matthias Totzeck, Ulrike B. Hendgen-Cotta, Brent A. French, Tienush Rassaf

**Affiliations:** ^1^Department of Cardiology and Department of Angiology, West German Heart and Vascular Center, Medical Faculty, University Hospital Essen, Essen 45147, Germany; ^1^Department of Biomedical Engineering, University of Virginia, Charlottesville, VA 22903, USA

**Keywords:** remote ischemic preconditiong, myocardial ischemia/reperfusion, plasma transfer, laser Doppler perfusion imaging, magnetic resonance imaging

## Abstract

Although urgently needed in clinical practice, a cardioprotective therapeutic approach against myocardial ischemia/reperfusion injury remains to be established. Remote ischemic preconditioning (rIPC) and ischemic preconditioning (IPC) represent promising tools comprising three entities: the generation of a protective signal, the transfer of the signal to the target organ, and the response to the transferred signal resulting in cardioprotection. However, in light of recent scientific advances, many controversies arise regarding the efficacy of the underlying signaling. We here show methods for the generation of the signaling cascade by rIPC as well as IPC in a mouse model for *in vivo* myocardial ischemia/reperfusion injury using highly reproducible approaches. This is accomplished by taking advantage of easily applicable preconditioning strategies compatible with the clinical setting. We describe methods for using laser Doppler perfusion imaging to monitor the cessation and recovery of perfusion in real time. The effects of preconditioning on cardiac function can also be assessed using ultrasound or magnetic resonance imaging approaches. On a cellular level, we confirm how tissue injury can be monitored using histological assessment of infarct size in conjunction with immunohistochemistry to assess both aspects in a single specimen. Finally, we outline, how the rIPC-associated signaling can be transferred to the target cell *via* conservation of the signal in the humoral (blood) compartment. This compilation of experimental protocols including a conditioning regimen comparable to the clinical setting should proof useful to both beginners and experts in the field of myocardial infarction, supplying information for the detailed procedures as well as troubleshooting guides.

## BACKGROUND

With 17.5 million (3 out of every 10 deaths) disease-related victims in 2012, ischemic heart disease and particularly acute myocardial infarction constitutes the leading cause of death worldwide. Prototypical myocardial infarction occurs as a result of arteriosclerotic plaque rupture and thrombus formation [1]. Occlusion of the corresponding coronary artery causes a cessation of blood, in particular oxygen (O_2_) supply to the area at risk (AAR). The treatment of choice is the restoration of vessel patency. A timely reperfusion regimen, preferably by coronary intervention, is the recommended therapy but the rapid restoration of blood and particularly O_2_ supply imposes additional reperfusion injury upon the heart with no current treatment available. Experiments targeting the mediators of this so-called ischemia/reperfusion (I/R) injury have provided novel therapeutic approaches. However, none of these strategies have yet been successfully implemented into clinical practice. The robust cardioprotective effect of ischemic conditioning, as documented in animal models, certainly warrants more innovative investigations both at the experimental and clinical levels [2,3].

**Figure 1.**
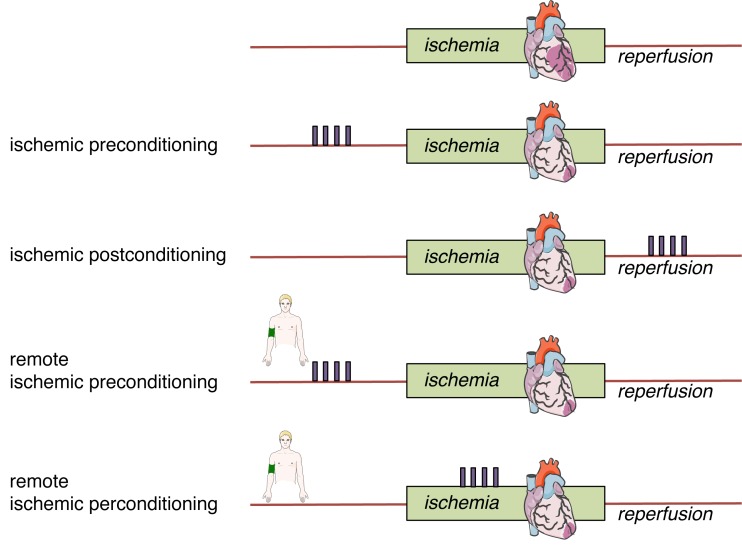
**Schematic outline of several conditioning regimen.** Myocardial I/R injury can be modulated by application of different ischemic conditioning approaches. Ischemic preconditioning and postconditioning relate to short non-deleterious phases of sublethal ischemia applied to the index organ (in this case the heart) before or after the main ischemia (row two and three). This can be conducted experimentally by arterial ligation and suture release in a repetitive manner and leads to reduction in infarct sizes. Remote ischemic conditioning is applied to a heart-distant organ, in this case the upper extremity through blood pressure cuff in- and deflations (row four). The stimulus can be applied prior to the heart (main) ischemia or—in case of perconditioning (row five)—during the main ischemia. Typically in all regimens 3–4 cylces of short non-deleterious conditioning ischemic events should be performed. This is typically 5 min long each. I/R: ischemia/reperfusion.

The term ‘ischemic conditioning’ refers to the priming of the tissue at risk with short non-deleterious and repetitive phases of sublethal ischemia [4]. In the scope of myocardial I/R, this renders the cardiomyocyte capable of protecting itself. The ischemic conditioning stimulus can be effectively applied before, during or after the index (main) ischemia (pre-, per-, postconditioning, **Fig. 1**) [4-9]. Interestingly, the ischemic stimulus does not need necessarily to be applied to the index organ, but can also be executed at a remote site, *e.g.*, the arm (**Fig. 1**). Conceptually, three components of remote ischemic preconditioning (rIPC) can be distinguished: the signal generation, the transfer of the signal to the target organ, and the response to the transferred signal resulting in cardioprotection. The underlying mechanisms are complex and remain to date incompletely resolved [9,10].

Many previous studies have used surgical ligation of the femoral vessels with or without discontinuation of the femoral nerve [11] to induce preconditioning at remote sites. This invasive approach was further combined with mere visual control of ischemia and reperfusion success in the hindlimb without the benefit of objective and reliable quantitative measurement methods.

The unique potential of translating rIPC to the clinical setting derives from its universal availability and non-invasive nature. A recent meta-analysis of current trials showed a clear effect of rIPC on clinical outcomes [12]. However, not all studies have concordantly reported beneficial effects from rIPC, and some study populations did not show any infarct-sparing effects. Therefore, animal models remain the system of choice for molecular studies into the underlying signaling mechanisms in rIPC with particular importance for mouse models given the availability of knockout specimen. The ideal rIPC stimulus in animal models should mirror the maneuver in patients, thus avoiding the pitfalls of blood loss, inflammation and *e.g.*, pain as induced by surgical techniques.

We have developed a clinically-relevant, non-invasive model of rIPC as translational bridge that closely mimics the approach in humans which avoids the caveats of previous approaches. We use a small occluder, which is placed around the right upper hindlimb, permitting a complete arrest of hindlimb perfusion. This occluder allows for an application of pressures after having been placed tightly around the hindlimb. Inflation will take place on the inner part of the occluder, while the shape and conformation of the outer part remain stable (see blood pressure cuffs in clinical routine). Importantly, this method avoids potential vascular and tissue damage while applying regulated pressure as required. In such studies, the efficacy of the ischemic episodes must be monitored to ensure reproducibility. We therefore established laser Doppler perfusion imaging (LDPI) measurements for the assessment of rIPC on hindlimb perfusion. This is a delicate issue as several circumstances influence the LDPI signal (*e.g.*, the presence or absence of hair, the type of anesthesia, body temperature and position). We have therefore placed particular emphasis on the exact positioning of the laser beam and the depilatory measures as well as the placement/fixation of the mouse during LDPI measurements. Finally, combining LDPI with a manometer for pressure application allows for the determination of systolic blood pressures in mice in parallel to the rIPC maneuver.

Following the outline of our *in vivo* rIPC model we put a particular emphasis on the evaluation of a blood-borne protective factor released by rIPC to induce a cardioprotective signaling. In our protocol, we mainly rely on an open-chest I/R model, which we consider to be technically less challenging than other optional approaches including the so-called closed-chest models. However, the latter also represents an alternative to the proposed technique. We have provided a list including (dis-)advantages of the different models in **Table 1**.

Studies in porcine models using heart transplants showed that rIPC remains effective in these denervated specimens. If rIPC released a protective substances distributed throughout the circulation then transfer of blood or plasma from conditioned specimen to unconditioned recipients should recapitulate cardioprotection. We therefore present a novel method to assess how transfer of plasma especially from human conditioned donors to mouse models (*e.g.*, with genetic deletion of target proteins) could be performed. This is a very effective method to assess the signaling in rIPC and to evaluate whether certain patients will benefit from rIPC while others do not.

**Figure 2.**
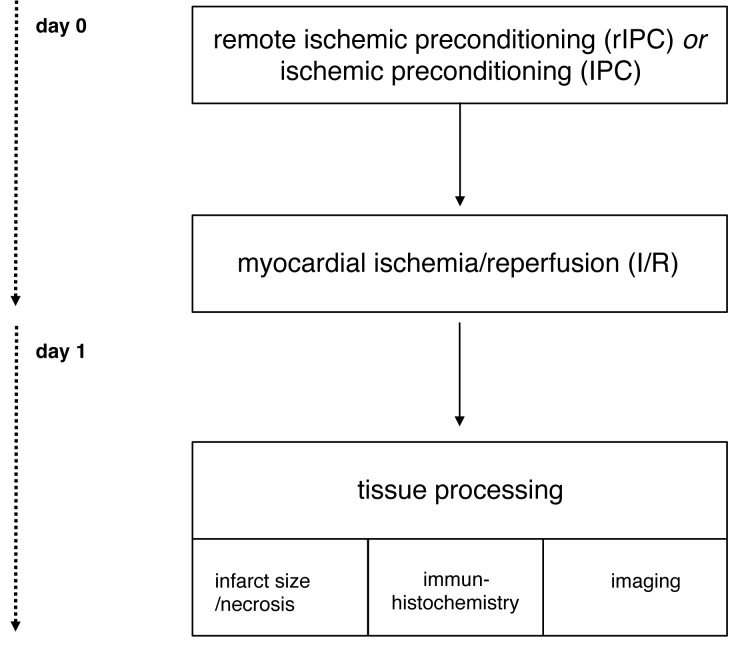
**Timeline of the modules performed during rIPC and IPC studies of the heart.** While modules one and two (rIPC/IPC and myocardial I/R, respectively) are typically performed on day 0 with an approximate duration of 1.5 h, module three comprising different aspects of measuring cardiac function and tissue processing are performed at day 1 in our protocol. Transfer experiments can be performed at day 0 and are not included in the schema. Tissue processing can include any treatment necessary to evaluate the parameter of choice. However, we here focus on three aspects: infarct size, immunohistochemistry or imaging techniques US and MRI. rIPC: remote ischemic precondition; US: ultrasound: MRI: magnetic resonance spectroscopy.

Based on recent submissions, we here describe a modular approach to our protocols (**Fig. 2**). It consists of five modules: (1) A non-invasive preconditioning procedure for rIPC checked by LDPI technique to establish reproducibility [9,13]; (2) Open-chest myocardial I/R *in vivo* protocol complemented by a short outline of preconditioning of the index organ (IPC); (3) Assessment of cardiac function by ultrasound (US) or magnetic resonance imaging (MRI) approaches; (4) Post I/R tissue processing for monitoring infarct size by non-invasive late-gadolinium enhanced (LGE) MRI or by staining the explanted and sectioned hearts with 2,3,5-triphenyltetrazolium chloride (TTC) and Evans blue dye; and (5) Plasma transfer experiments to assess the characteristics of the humoral factors released by rIPC to induce a protective cellular signal at a distance.

## MATERIALS

### Animals

Mice are kept at the local animal house in a temperature- and humidity-controlled room under a 12 h light-dark cycle with free access to food and water. All experimental procedures described here were approved by the Landesamt für Natur und Umweltschutz, Recklinghausen, Nordrhein-Westfalen, Germany, and the Institutional Animal Care and Use Committee and conformed to the ‘Guide for the Care and Use of Laboratory Animals’ (NIH Publication 85–23, revised 1985).

**NOTES**: Experiments must comply with national and institutional regulations concerning the use of animals for research purposes and permissions to carry out experiments have to be obtained.

**HINTS**: Generally, myocardial I/R injury as outlined here can be performed in all mouse models. In our laboratory, we use adult mice at a minimum of 12 ± 3 weeks of age and with an average minimum weight of 25 ± 4 g. Using younger or lighter animals may pose difficulties. This largely results from the technical difficulties in identifying the left and right coronary artery (LCA) in small specimen. Taking advantage of a wide variety of knockout models, we note that in genetically modified mice that affect vascular beds (*e.g.*, eNOS-deficient mice [14]) the correct positioning of the coronary suture can be particularly delicate.

**Table 1.** Advantages and disadvantages of available *in vivo* mouse infarction models.

*In vivo* model	Advantages	Disadvantages
Open chest technique	• Robust experimental evidence/data, reproducibility • Visual and ECG control of ligation success	• Surgery time of 20 min • Requires mechanical ventilation
Permanent ligation	• Less technically challenging	• Lack of reperfusion (I/R injury not present) • Large infarction
Closed-chest technique	• Closed-conditions	• Technically challenging • Post-surgery defective healing • Success of infarction only via ECG
Complete externalization of the heart technique	• Fast method (min) • Omits mechanical ventilation	• Very short phase of global hypoxia during short phase of heart externalization (approx. 8 s) • Requires advanced surgical skills and animal handling • Difficulties in handling/detecting pneumothoraces

### Human materials

Our transfer experiments further included plasma from humans.

**NOTES:** Any study in humans requires approval by the institutional ethics committee and all participants must have given written consent to the study protocol in advance.

### Reagents

• Acetic acid (Sigma-Aldrich, Seelze, Germany, cat. # 34255)
• Aquasonic Clear Ultrasound Gel (Parker Laboratories Inc, Fairfield, NJ, USA)
• Buprenorphine (Essex Pharma, München, Deutschland, cat. # PZN 0345928)
• CaCl_2_ (Sigma-Aldrich, Seelze, Germany, cat. # C1016)
• Carbogen (Air Liquide, Oberhausen, Germany)
• Evans blue (Sigma-Aldrich, Seelze, Germany, cat. # E2129)
• Glucose (Sigma-Aldrich, Seelze, Germany, cat. # G5767)
• Hair removing crème, Veet (Reckitt Benckiser Deutschland GmbH, Heidelberg, Germany)
• Heparin (70–100 IE; ratiopharm, Ulm, Germany)
• Isoflurane (Abbvie, Ludwigshafen, Germany, cat. # B506).
**CAUTION**: Prolonged exposure to isoflurane potentially causes central nervous system depression. An exhaust system to eliminate excessive isoflurane needs to be operational during the surgery.
• KCl (Sigma-Aldrich, Seelze, Germany, cat. # P9333)
• KH_2_PO_4_ (Sigma-Aldrich, Seelze, Germany, cat. # 1551139)
• MgSO_4_ (Sigma-Aldrich, Seelze, Germany, cat. # M7506)
• Mounting medium ‘Vitro Clud’ (R. Langenbrinck, Emmendingen, Germany, cat. # 04-0001)
• Nair hair removal lotion (Church & Dwight Co., Inc, Ewing, NJ, USA)
• NaCl (Carl Roth, Karlsruhe, Germany, cat. # 3957.3)
• NaHCO_3_ (VWR, Darmstadt, Germany, cat. # 27.775.293)
• NaH_2_PO_4_ (Sigma-Aldrich, Seelze, Germany, cat. # 55011)
• Na_2_HPO_4_ (Carl Roth, Karlsruhe, Germany, cat. # T876.2)
• Octenisept (Schülke & Mayr, Norderstedt, Germany, cat. # 173704)
• Omniscan gadolinium-based MRI contrast (Nycomed Amersham, Oslo, Norway)
• Oxygen, medical (Linde, Unterschleissheim, Germany)
• Phosphate buffered saline without Ca^2+^/Mg^2+^ (Sigma-Aldrich, Seelze, Germany, cat. # D8537)
• Rompune (aniMedica, Senden-Bösensell, Germany)
• Sodium pyruvate (Sigma-Aldrich, Seelze, Germany, cat. # P8574)
• Ketamine (Pfizer, Berlin, Germany, cat. # NO1AXO3)
• 2,3,5-triphenyltetrazolium chloride (Sigma-Aldrich, Seelze, Germany, cat. # 93140)
**Recipes**
**TTC staining solution:** Prepare two phosphate buffer solutions (buffer 1 and buffer 2) by solving 14.2 g Na_2_HPO_3_ in 1 L ultrapure water (0.1 M, buffer 1) and 6 g NaH_2_PO_4_ in 0.5 L ultrapure water (0.1 M, buffer 2). Calculate 10 ml final TTC solution per heart. For this, add 7.44 ml of buffer 1 to 2.26 ml of buffer 2. Measure pH (target pH: 7.4). Add buffer 1 to decrease or buffer 2 to increase pH if incompliant with target value. Finally, add 100 mg of TTC to 10 ml of the mixture composed of buffer 1 and buffer 2.
**CAUTION:** This solution should be prepared freshly every day and stored in the dark.
**Krebs-Henseleit buffer:** The following procedure refers to a buffer volume of 2 L, which is to our experience sufficient for at least three to four isolated heart preparations. Final concentrations in 2 L buffer are given in brackets: Add in the following order 13.8 g NaCl (118 mM), 0.7 g KCl (4.7 mM), 0.4 g MgSO_4_ (0.8 mM), 4.18 g NaHCO_3_ (25 mM), 0.32 g KH_2_PO_4_ (1.2 mM), 1.8 g glucose (5 mM) and 0.44 g sodium pyruvate (1.1 mM) to 2 L of ultrapure water. When completely solved, gas buffer with carbogen. After a minimum of 15–20 min, add CaCl_2_ (0.74 g; 2.5 mM final concentration) to gassed buffer. Filter buffer using a 0.45 µM filter.
**CAUTION:** This solution must be prepared freshly every day.

### Equipment

• Milli-Q Advantage A10, ultrapure water system (Merck Chemicals, Schwalbach, Germany
• Heated small animal operating table usually operated at 37°C–38°C (Hugo Sachs Elektronik, March-Hugstetten, Germany, cat. # 50-1247)
• Mouse ventilator ‘Minivent’ (Hugo Sachs Elektronik, March-Hugstetten, Germany, cat. # 73-0044)
• Ventilation system (gases and isoflurane) (UNO Roestvaststaal BV, AA Zevenaar, Netherlands, cat. # 180000206, 180000002, 180000004, 180000005, 180000010)
• Suspension device (Intubation Aid) (UNO Roestvaststaal BV, AA Zevenaar, Netherlands, cat. # 180000014)
• Occluder (OC4, OC 5, OC6, OC8) Kent Scientific, Connecticut, USA)
• Manometer (KAL 84, Halstrup Walcher, Kirchzarten, Germany, cat. # 9095.0074)
• Stereotactic microscope (Leica Mb 5; Leica MZG; Wetzlar, Germany)
• Camera (Hitachi HV C20AMP, Erkrath, Germany)
• Langendorff apparatus Basic IH-SR with aortic cannula and cannulation box (Hugo Sachs Elektronik, March-Hugstetten, Germany, cat. # 73-4343, 73-2806, 73-4019, 73-4021, 73-0129)
• Langendorff apparatus System Isoheart Software (Hugo Sachs Elektronik, March-Hugstetten, Germany, cat. # 73-4039)
• ClinScan 70/20 Superconducting Magnet System, including gradient system B-GA 12S2, MAGNETOM Avanto/Trio digital radio frequency system, syngo MR B17 acquisition software and animal holder (Bruker BioSpin MRI GmbH, Ettlingen, Germany)
• Mouse body quadrature volume radiofrequency coil (Bruker BioSpin MRI GmbH, Ettlingen, Germany)
• Model 1030 Monitoring & Gating System (Small Animal Instruments, Inc, Stony Brook, NY, USA)
• Vevo 2100 High Resolution Ultrasound System with MS400 solid-state, linear array transducer and imaging station (FUJIFILM VisualSonics, Inc, Toronto, Ontario, Canada)
• Cooling plate (self-made)
• Pacemaker electrode (stimulator C type) (Hugo Sachs Elektronik, March-Hugstetten, Germany, cat. # 73-3713, 73-0160)
• Infusion pump (Harvard Pump 11 Plus dual syringe, Harvard Apparatus, cat. # 70-4501)
• Polyethersulfone membrane filters 0.45 µm (Sartorius, Gottingen, Germany, cat. # 15406
• Laser Doppler perfusion imager (Perimed, PeriScan PIM 3, Software LDPIwin 3.1.2, Järfälla, Sweden)
• Thermomixer (VWR, Darmstadt, Germany, cat. # 460-0223)
• Infarction software Diskus (Hilgers, Königswinter, Germany)
• Wella Contura Professional Hair Trimmer (Wella, type HS60)
• Needle holder (Aesculap; Braun, Melsungen, Germany, cat. # FD233R)
• 24G or 26G sterile needles (BD Microlance 3, BD Biosciences)
• 21G venous cannulas (Braun, Melsungen, Germany)
• Syringes (sterile 1 ml; perfusor 20 ml; Braun, Melsungen, Germany)
• 4-0, 5-0, 6-0 prolene suture (Ethicon; Johnson & Johnson, Norderstedt, Germany, cat. # EH 7814, EH 7231, EH 7478)
• Small and micro-scissors (FST, Essen, Germany, cat. # 14059-11, 15007-08, 14064-11)
• Forceps (FST, Essen, Germany, cat. # 11203-25, 11069-08, 11616-15, 11506-12, 11051-10; Aesculap Braun, Melsungen, Germany, cat. # BN731R, BD 311R)
• Retraction Kit (FST, Essen, Germany, cat. # 18200-20)
• Small silicon tube (Reichelt Chemietechnik, Heidelberg, Germany)
• Gauze pads (Hartmann, Heidenheim, Germany, cat. # 407835)
• Glass slides (Carl Roth, Karlsruhe, Germany, cat. # K532.1)
• Centrisart tube (Sartorius, Göttingen, Germany, cat. # 13249)
• Neonatal MR-compatible electrocardiogram (ECG) electrodes (Vermed, Bellows Falls, VT, USA)

## PROCEDURE

### Induction of anesthesia, intubation, mechanical ventilation • TIMING 10–12 min

**Figure 3.**
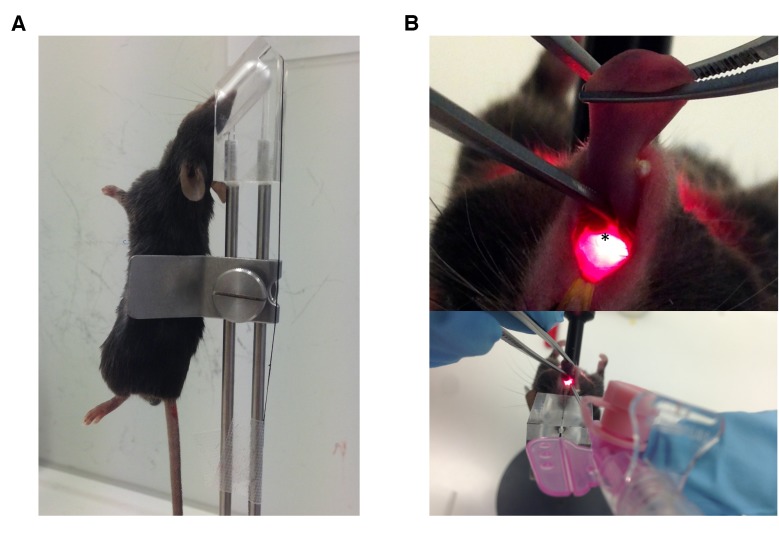
**Intubation procedure. A.** Use a suspension device and suspend the mice with the upper front teeth. **B.** Cold light is very helpful when identifying the larynx. Place light against the middle of the trachea and pull the tongue out (upper panel). The asterisk marks the larynx in bright light. One-way sterile venous cannulas are optimal tracheal tubes for mice. Lower panel shows intubation procedure.

**HINTS:** For all modules, mice are under deep sedation over the whole specific procedures. We use tracheal intubation rather than tracheotomy as outlined in other protocols [15]. In our hands this provides a much better handling of mice in the wake-up period.
1. The mice are kept at the local animal house and will be transferred to the operation room on the day of surgery.
**CRITICAL STEP:** New mice shipped from suppliers should be allowed to become accustomed to the environment (approximately seven days). Check mice for wounds, healing defects, and infections, as an activation of the immune system can significantly influence I/R outcomes.
2. Set-up the surgical heating plate, ventilation system (gases and isoflurane), anesthetics and materials.
3. Record the following: mouse weight, age, strain, date and time of experiments. Complete one surgery protocol sheet per mouse.
4. Calculate the necessary dose for ketamine and rompune required for deep anesthesia and buprenorphine for analgesia (ketamine 100 mg/kg body weight, rompune 10 mg/kg, buprenorphine 0.05–1 mg/kg body weight).
**NOTES:** Drugs used for sedation and analgesia as well as animal handling procedures must comply with national and regional laws and will require permission from the necessary committee. Anesthesia depth was confirmed by failure of limb withdrawal upon toe pinching. (Order of anesthesia induction, see supplementary **Fig. S1**)
5. Fill a 1 ml sterile syringe with the calculated amount of anesthetics. Inject the drugs carefully intraperitoneally (*i.p.*) using a 24G or 26G sterile needle. Wait for the cessation of any spontaneous movement (duration 8–10 min), spontaneous breathing will persist.
6. Remove the chest hair by using a small animal shaving machine. This should take place with distance to the surgical site to avoid infections. Hindlimb hair removal is also mandatory for LDPI studies.
7. Attach the mouse with the front teeth to the suspension device (**Fig. 3A**) and position cold light in front of the trachea.
**HINTS**: Use small forceps to retract tongue out of the oral cavity to one side of the mouth, locate the illuminated area within the trachea and use a small tube to conduct intubation (21G venous cannulas represent excellent tubes for mouse intubation, **Fig. 3B**). Detach the mouse carefully from the suspension device, place it onto the surgical plate and attach the tube to the ventilation system. Intubation success can be visualized when the chest and not the abdomen moves with mechanical breathing. In some respirators, barometers may also indicate correct tube positioning by measuring spontaneous breathing. We set our respirators to a breathings frequency of 140/min and a tidal volume of 100–120 µl. Please refer to the manufacturer’s instruction for correct settings as this relies on the respirator’s specifications and the tubing.
8. Maintain anesthesia using isoflurane (1.2–2 Vol%) in conjunction with a gas mixture of 0.2 L/min O_2_ and 0.2 L/min compressed air. Fix the tube and the extremities using adhesive tape. For help in case of difficult intubation see troubleshooting.

### rIPC on the hindlimb • TIMING 45–50 min

**Figure 4.**
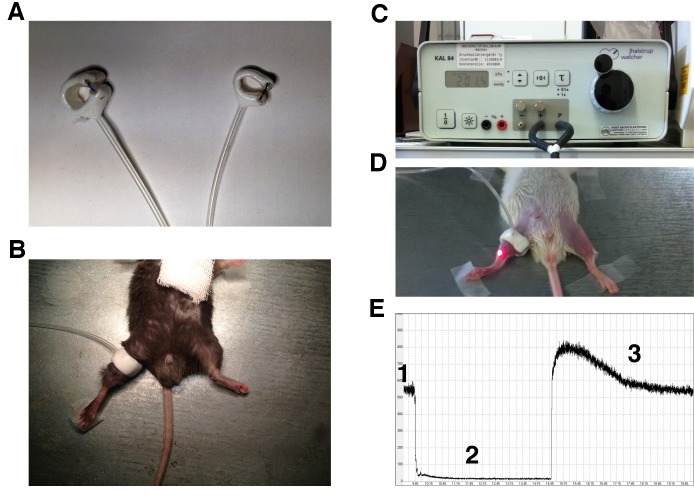
**Remote ischemic preconditioning using a non-invasive occluder-based LDPI controlled approach. A.** Set of occluders at different sizes (left 8 mm inner diameter, right 5 mm). **B.** The occluder is placed around the hindlimb of a C57BL/6 mouse, which is fixed with adhesives on a surgical heating plate. Procedure can be compared to placement of blood pressure cuffs in humans, as used in conditioning studies. **C.** The cuff tubing is the attached to the manometer, which can precisely measure and apply pressure to inflate the cuffs inner diameter. The laser Doppler is placed above the mouse and the laser beam place on the lower hindlimb. **D.** Picture shows LDPI on a NMRI mouse. Hair was carefully removed on the lower legs to allow full laser penetration. **E.** Typical LDPI recording with 3 phases: baseline perfusion (1); arrest of perfusion when inflation the cuff to 200 mmHg or above (2) and reperfusion phase (3). Notice the increase in reperfusion above baseline levels, which is an indication of hyperemia in reperfusion. Off note, reperfusion as measure by LDPI has its onset at the systolic blood pressure. Careful examination of perfusion in relation to pressure on the manometer could additionally supply these pressures in the individual mouse. rIPC: remote ischemic reconditioning; LDPI: laser Doppler perfusion imaging; NMRI: Naval medical research institute.

**NOTES:** rIPC originates a signal in the remote organ that is readily transferred to the target organs. The remote site can be a solid organ, a specific vascular bed or an extremity. For clinical translation, leg or arm rIPC are certainly the most favorable approaches. Inflation of a small occluder device—in analogous to blood pressure cuffs in patients—is a simple approach to activate protective signaling. If an exclusion of the afferent nervous system is part of the experimental approach, excision of the femoral nerve prior to rIPC can be an advantageous approach.
9. Sufficient anesthesia is achieved when the withdrawal reflex of the hindlimb vanishes.
10. Deflate the occluder completely (**Fig. 4A**) and place it around the upper hindlimb (**Fig. 4B**). Be prepared by having different sizes of occluders available to ensure complete cessation of perfusion.
11. Attach the occluder *via* a tube to the manometer (**Fig. 4C**) and zero the system by opening it to room air.
**NOTES:** LDPI is a non-invasive technique to study the cessation and recovery of perfusion during occluder in- and deflation periods. Incomplete blood flow arrest in the rIPC ischemic phases may indicate *e.g.*, that the occluder size is too large in relation to the respective hindlimb dimensions (*e.g.*, hindlimbs in obese mice *vs*. standard littermates). Careful attention should be paid to the LDPI set-up and surrounding environmental conditions (see troubleshooting). Avoid changes in light intensity and quality during LDPI studies, adjust to the basal background threshold and do not modify hereafter. Keep and mark (if not conducted by the system’s software) the distance between leg and laser source (approximately 20 cm). Maintain heating plate temperature at 37°C–38°C throughout. Do not move the mouse. This again requires a sufficient depth of anesthesia. Troubleshooting for LDPI measurements is provided in the TROUBLESHOOTING section.
12. Re-fix the hindlimb carrying the occluder using adhesive tape. Position the LDPI to a region below the occluding device (*e.g.*, distal lower leg) with a small region of interest (**Fig. 4D**).
13. Begin with the recording of the LDPI measurements using the scanner in the Duplex mode at a sampling rate of 100 Hz, filter cut off frequency 1.25 Hz, and a threshold for light intensity of 0.3.
14. Start the rIPC protocol by inflation of the occluder. This will increase the inner diameter and causes an arrest of blood flow to the hindlimb. Typically, a pressure of 200–250 mmHg will be sufficient. If higher pressure must be applied, consider decreasing the diameter of the occluder. Likewise, if perfusion ceases below 120–150 mmHg, a larger occluding device may be helpful. After the ischemic phase of the rIPC stimulus (5 min) release the cuff pressure. This should result in a recovery of blood flow. Observe the pressure levels at which perfusion restarts. This is the systolic blood pressure and should be noted as such. Within the first 1–2 min, an increase of flow above baseline levels can be noted. This indicates hyperemic flow in the reperfusion phase (**Fig. 4E**) [9]. Due to its non-invasive nature, LDPI is by itself a semi-quantitative method. In this protocol, we take advantage of LDPI to measure baseline hindlimb perfusion, check whether cuff-inflation causes a complete cessation of blood flow and finally test whether reperfusion is achieved when releasing the pressure from the cuff. Units in the graph are therefore arbitrary units. We expect an increase of blood flow to 150%–220% of the baseline perfusion. Comparable values were obtained for humans [9].
15. Continue with the next phases of the rIPC protocol, *e.g.*, a total of 4 phases with 5 min of hindlimb ischemia and 5 min of reperfusion. This totals 40 min. Bear in mind that directly after the rIPC maneuver, many researches wish to continue with the main myocardial ischemia. Depending on the exact protocol, plan on immediately starting with surgery once the protocol is finished. Help from a second person in order to remove the LDPI/rIPC devices while the other person prepares for surgery may be helpful.

### Myocardial I/R including IPC • TIMING 45–90 min with or without IPC protocol

**Figure 5.**
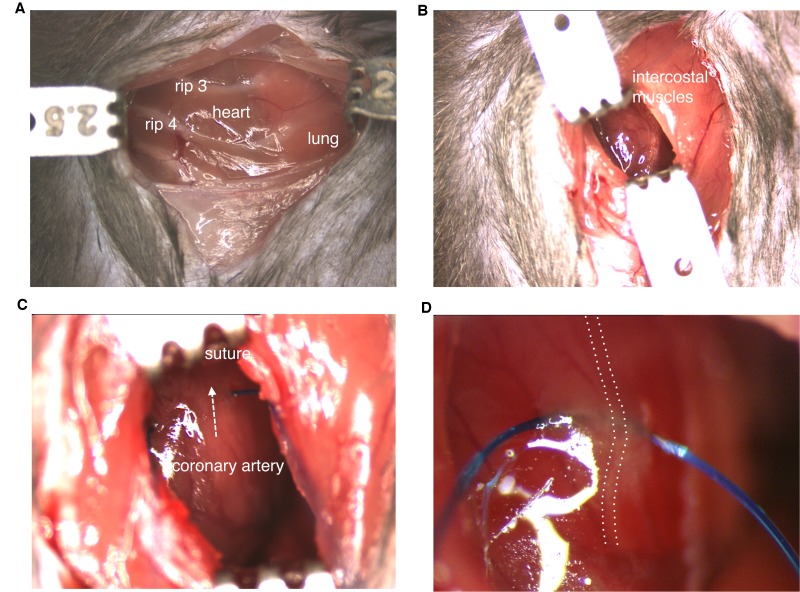
***In vivo* LCA ligation for the induction of I/R. A.** The thorax is opened (incision length 15–18 mm). The wound is held open by surgical hooks. Access to the heart is gained between third and fourth rib. **B.** The intercostal muscles are carefully prepared to gain access to the pericardium. **C.** After identification of the LCA, place the suture for IPC or myocardial I/R in a loop around LCA. **D.** Note that the LCA will appear white (scattered lines) while cardiac veins have a much thinner vessel walls and typically appear filled with blood. IPC: ischemic preconditioning; LCA: left coronary artery; I/R: ischemia/reperfusion.

**Figure 6.**
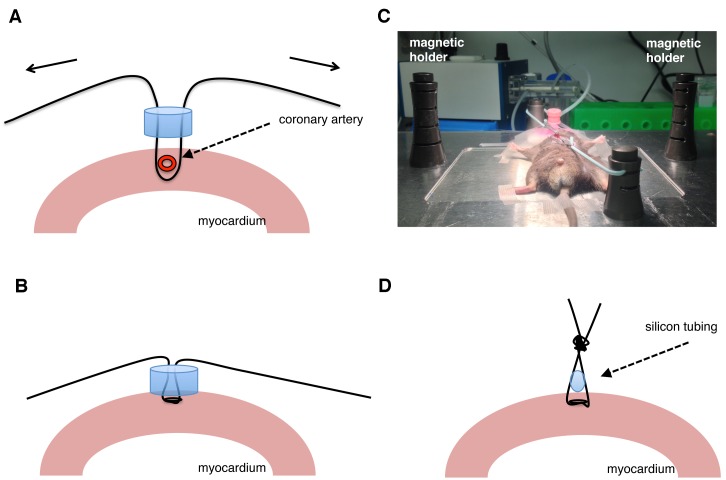
**Schematic drawing of coronary ligation set-up for IPC and ischemia. Pass suture through tube, then encircle LCA.** Pass the suture again through the tube. **A.** Arrows indicate direction of force to ligate LCA in the ischemic phases of the IPC protocol. **B.** Pulling the suture ends way from the mouse leads to closure of the LCA. **C.** Photograph showing magnetic holders to which the suture is attached during IPC. **D.** In contrast to IPC, during myocardial main I/R, a silicon tube is placed on the loop that encircles the LCA. Place a tight surgical knot on the silicon tube to ligate the LCA. IPC: ischemic preconditioning; LCA: left coronary artery.

**NOTES**: The open-chest myocardial I/R model exhibits certain advantages and disadvantages. It is certainly the most common *in vivo* I/R model and results (infarct sizes, arrhythmia, and cardiac enzyme levels) can be related to a vast amount of prior studies. However, extrapolating results from mouse to the clinical situation is difficult as the pathomechanism of infarction in patients is the spontaneous plaque rupture. Other *in vivo* infarction models include the permanent ligation technique, the closed-chest model [16], and an open-chest model with complete translocation of the heart outside the thorax [17].
While parts 9–15 of the protocol focus on rIPC, IPC is outlined in conjunction with the following sections on myocardial I/R because it involves the opening of the thorax.
16. Use antiseptics to minimize bacterial flora in the operation field. Wait for skin disinfectant to dry. The next steps should be conducted under a dissection microscope.
17. Perform skin incision using small surgical scissors. Cut from the middle of the sternum over the heart (heart beat can be localized by palpation prior to surgery) towards the left axilla. The total length of the incision should be 15–18 mm.
18. Use appropriate tweezers for a blunt preparation of the two muscle layers (intercostal) and two surgical hooks until ribs can be visualized.
19. Opening of the thorax will be performed between the third and fourth rib. Use micro-scissors for this part. Prepare the two intercostal muscle layers. Two surgical hooks placed at either side of the incision are used to increase the diameter of the thoracotomy (**Fig. 5A**). Gentle force on the hooks can be helpful to gain access to the pericardium (**Fig. 5B**).
20. Resect the pericardium above the heart. This avoids post-surgery pericardial effusion.
**HINTS**: Localize the LCA (**Fig. 5C** and **5D**). Use a 6-0 prolene suture to encircle the artery halfway from base to apex with a loop.
21. At this point of the protocol, continue either with IPC followed by main ischemia or go directly to Myocardial I/R.
  21.1. IPC
    21.1.1. Pass the suture through a small silicon tube. Attach the endings of the suture to a magnetic holder at either side of the mouse.
    21.1.2. Begin with IPC protocol by moving the magnetic holders simultaneously to the left and to the right away from the mouse, which will tighten the suture and occlude the coronary artery (**Fig. 6A-6C**).
    21.1.3. Observe whether the myocardium turns grey below the occluded artery.
    21.1.4. After this ischemic phase, release the suture by moving the magnetic holders towards the mouse and observe whether the myocardium returns to its red color.
    21.1.5. Finish the IPC protocol by conducting *e.g.*, a total of 4 cycles with 5 min of ischemia and 5 min of reperfusion per phase. Then continue with option B to perform the myocardial I/R.
  21.2. Myocardial I/R
**NOTES:** Mouse coronary arteries largely consist of two main arteries (left and right coronary artery). A clear separation into a circumflex artery and left anterior descendent (LAD) as compared to humans does not exist [18]. The ligation of a left wall myocardial territory therefore refers to occlusion of the LCA and not the LAD as noted in many previous publications.
    21.2.1. Position a small silicon tubing above the loop (**Fig. 6D**) and place a tight knot above the tube. This way of occluding the LCA remain tightened for a long time period but cannot be released as quickly as required for IPC regimens.
    21.2.2. Observe whether the myocardium turns grey distal to the knot.
    21.2.3. Release the surgical hooks slightly. Place a wet gauze pad on the operation site to avoid drying up.
    21.2.4. Continue ischemia for the desired time, *e.g.*, 30 min as frequently applied by our laboratory.
    21.2.5. Carefully observe the animal and the operation site for any immediate signs of acute heart failure (arrhythmia, drop in heart rate). Any of these signs should result in an immediate cessation of the procedure.
22. At the end of the IPC protocol followed by main ischemia (both IPC and Myocardial I/R) or option Myocardial I/R (main ischemia only) release the silicon tube to induce reperfusion. Observe the return of a more reddish color to the myocardium. Loosen the suture which will be left in place and retightened in the post-processing protocol.
23. Close the thorax cavity layer by layer using a 4-0 prolene suture. Begin with the ribs and re-approximate the ribs using a silk suture. Slightly increase tidal volume on your respirator (volume controlled respirator as performed by us) or increase inspiratory pressure (pressure controlled ventilation) depending on your respirator’s specifications. This leads to a fully inflation of the lung. Then tighten knots between the ribs, adapt muscle layers (2-3 5-0 prolene sutures) and perform skin closure (4-5 5-0 prolene sutures).
24. Switch off the isoflurane line. After 2–3 min, observe whether the mouse regains consciousness and starts spontaneous breathing. Once spontaneous breathing is sufficient, extubate the mouse. Place the animal in a clean and warm cage and inject 0.05–1 mg/kg buprenorphine subcutaneously for analgesia every 8 h.

**PAUSE POINT**

### Non-invasive measurement of cardiac function by US or MRI • TIMING 15–60 min

**NOTES:** Non-invasive determinations of left ventricular ejection fraction (LVEF) are best calculated from accurate measurements of LV end-systolic volume (LVESV) and LV end-diastolic volume (LVEDV). The two most common imaging platforms used for making these measurements in mice are high-frequency US and high-field MRI. Each modality has its pros and cons, so the best choice is largely determined by the instrumentation, expertise and staff support available at a particular institution. Conventional clinical approaches to echocardiography typically use a limited number of long- and short-axis image planes to make rough estimates of LV volumes. However, the accuracy of the LV volumetric determinations made by US can be improved to rival that of MRI by adopting the MRI convention of analyzing 6–8 contiguous, 1mm-thick, short-axis image slices capturing the entire LV from base to apex. This typically involves immobilizing the transducer and using a micromanipulator to translate the stage (or transducer) in precise 1mm increments along the long-axis of the LV. Going beyond the global measure of EF, both US and MRI can be used to determine regional cardiac function through the measurement of myocardial strain *via* speckle-tracking and DENSE, respectively [19].

25. Cardiac imaging is typically performed 24 or 48 h post-reperfusion, allowing the mouse adequate time to recover from infarction, and yet imaging prior to the onset of significant LV remodeling. The animal is lightly anesthetized with isoflurane (as described above) and placed on a warm surface during preparation for imaging.
  25.1. For US imaging, this involves shaving the chest region over the heart with a hair trimmer, then applying Nair for the minimum amount of time necessary to remove any remaining hair from the region to be imaged. Particular care should be taken to rinse the area treated with Nair thoroughly to prevent burns from the active ingredient (sodium hydroxide). Care must also be taken to remove any air bubbles from the US gel (by centrifugation, if necessary) to avoid the image artefacts that inevitably result from imperfect acoustic coupling by the US gel.
  25.2. For MRI, the same combination of trimming and Nair treatment is used to remove all hair from 2-3 fore- and hindlimbs for the placement of neonatal ECG electrodes. The self-stick, MR-compatible ECG electrodes are then applied to the limbs to achieve maximal surface contact and secured with adhesive tape.
  25.3. Once prepared, mice are transferred from the prep area to the imaging platform, whether it be a heated stage with integrated ECG electrodes (for US) or a radiofrequency (RF) coil to be positioned at the isocenter of the MRI scanner. Regardless of imaging modality, particular care must be taken to actively monitor and maintain core body temperature at 37°C ± 0.5°C since both since both heart rate and cardiac function are dependent on temperature
  25.4. Under light isoflurane anesthesia (1.2%–1.5% in medical oxygen), with a core body temperature of 37°C ± 0.5°C and a heart rate of 475–575 bpm, non-invasive imaging is then performed to capture 1mm-thick, short-axis images of the cardiac cycle at 1mm increments from base to apex according to manufacturer’s instructions. Supplementary instructions are available on the manufacturer’s websites.
  25.5. If high-resolution maps of regional contractile function are desired, then additional imaging time may be needed to acquire US images at high temporal resolution (50–200 frames/cardiac cycle) or, in the case of MRI, by acquiring additional DENSE [19] or HARP [20] data to track myocardial motion over the cardiac cycle.
  25.6. Images are typically stored in DICOM format for the analysis of LVESV, LVEDV and LVEF using post-processing software provided by the respective vendor. For MR image datasets, alternative methods of image analysis include free online resources such as Segment (http://medviso.com/products/segment).

### Tissue processing to assess infarct size after rIPC/IPC • TIMING 2.5 h

**Figure 7.**
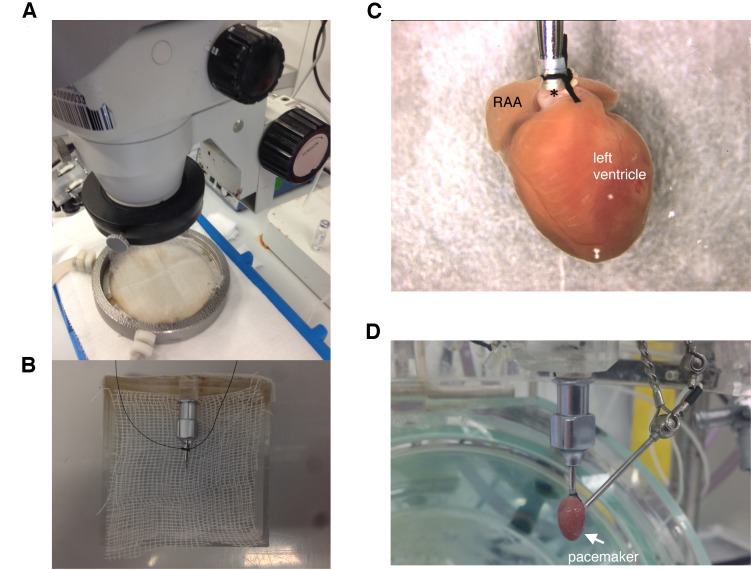
Excision of the heart. **A.** Cooling plate filled with ice cold NaCl 0.9%, which is used for preparation of the heart for cannulation. **B.** Cannulation chamber with metal cannula and preformed suture loop. **C.** Aorta is cannulated and suture closed to hold heart in place. Asterisk indicates the end of the aorta and the suspected area of the aortic valve and RAA indicates right atrial appendage. **D.** Attach cannulated heart to the langendorff system.

26. After Step 25, an intraperitoneal catheter of PE10 tubing is typically positioned in the mouse prior to insertion into the RF coil. The PE10 tubing is then routed out of the bore of the MRI scanner and connected to an insulin syringe so that Gd-based contrast agent (Omniscan) can be infused when needed as a bolus dose of 0.5 mmol/kg. LGE imaging is initiated 15–20 min later using either a cine-FLASH acquisition at an elevated flip angle [21] or an inversion recovery pulse sequence [22].
  26.1. *By MRI.* In the event that MRI is used to determine cardiac function (Step 25), investigators have the option of non-invasively determining the size and location of myocardial infarction using LGE MRI [21]. Alternatively, myocardial injury can be measured from circulating levels of cardiac enzymes (particularly troponin). In this context, recent studies in small rodents have showed that troponin levels peak at 24 h in rats while the maximum in mice can be detected 24–48 h after the onset of ischemia [23]. In case, troponin blood levels are considered, attention should there be paid to the exact timing of blood sampling for comparison. Alternatively, troponin level follow-up studies at consecutive time-points (*e.g.*, every 6–12 h) can be performed thus providing an area under curve result.
  26.2. *By TTC staining.* For determination of infarct sizes (lethal I/R injury), allow a reperfusion period of 24 h. However, reperfusion may be shorter or longer depending on the respective scientific question.
27. Apply narcotics to the mouse (Steps 4 and 5), but add 70–100 IE unfractionated heparin per kg bodyweight as *i.p.* bolus to establish complete anticoagulation. Wait for deep anesthesia.
28. Fix the mouse on the surgery plate and quickly perform the sternotomy. Excise the heart within 30 s and transfer it to the cooling plate filled with 4°C ice-cold sodium chloride (NaCl) 0.9% or a buffer of choice (**Fig. 7A**). The heart will immediately stop beating. The use of Ca^2+^ free buffers may further help.
29. Prepare the heart for aortic cannulation.
**CAUTION:** The following preparation of the heart must be conducted meticulously to preserve the integrity of the heart and the aorta, but should be completed as soon as possible with a maximum duration of 3–4 min. A thorough knowledge of the mammalian heart anatomy is required. Begin by dissecting the lungs (if obtained from the thorax *en block*) and thymus. Continue with the adipose tissue surrounding the large vessels. Localize the aorta, of which a remainder of 4–5 mm in length should be left for cannulation. Transfer heart to cannulation box.
**HINTS:** Using two forceps, take hold of the aorta, pull over the metal cannula and fix using a silk suture (**Fig. 7B**). The cannula must end distal to the aortic valve (**Fig. 7C**). Avoid any air in the cannula, aorta and left ventricle.
30. Attach the cannula with the isolated heart to a syringe filled with NaCl 0.9%. Gently perfuse the heart free of blood.
31. Re-occlude the LCA using a 6-0 suture. Alternatively, the investigator may wish to leave the original suture in place after myocardial *in vivo* I/R. This is then re-occluded. Both are practical alternatives.
32. Attach the cannula to the syringe filled with 1% Evans blue and flush the heart gently with dye. Alternatively, staining with 5% Evans blue can be performed if 1% does not provide sufficient results. Furthermore, *in vivo* perfusion with dye is an alternative to the proposed procedure.
33. Detach the heart from the cannula and wrap in cling film. Transfer the heart to the −20°C freezer and let the heart freeze (duration 60 min).
34. Unwrap the heart and cut it into 2 mm slices while holding it with forceps. For cutting, razor blades or microtome blades should be used.
35. Transfer the slices to a vial containing preheated TTC (37°C). Incubate for 5 min at 37°C on a shaker.
36. Obtain photos of the slices and label the photos (order of slices). Then use a precision scale and record the weight of each slide.
37. Use an infarction software to determine the size of the left ventricle, AAR (area of the left ventricle not stained by Evans blue) and infarct area (white as determined by TTC). Use software algorithm to assess the AAR/left ventricle (quality control) and infarct size per AAR based on planimetry in all slices. (Refer to ANTICIPATED RESULTS section for typical results, *e.g.*, with or without rIPC or IPC treatment).

### Transfer plasma preparation from donors e.g., humans • TIMING 60–80 min

**NOTES:** This is naturally an *ex vivo* experiment with certain disadvantages, *e.g.*, the lack of whole blood preparations and an intact circulation. However, it is an elegant bio-assay to test a distinct signaling elicted by rIPC.
**HINTS**: Isolated mouse hearts can be perfused with ultrafiltrated transfer plasma in a retrograde perfusion system according to langendorff [9,24-26] to test whether protective substances in the donor plasma are effective in the recipient heart. Transfusion of biological fluids and in particular plasma to isolated mouse hearts operated in a langendorff mode is difficult to achieve [27] particularly when involving interspecies samples (human to mice). The transfusion of complete plasma/serum or whole blood preparations can potentially initiate deleterious immunogenic responses and result in vascular obstruction in the recipient heart and immediate heart failure. Therefore coronary flow (perfusate flow) should be monitored continuously while any significant changes in perfusion can indicate heart failure. Furthermore pacing must be continued throughout. Failure of pacing may also be an indicator for heart failure. Cut off filters can be used to obtain a filtrate without proteins that are larger than 10–20 kDa while the size of protective factors is expected to be smaller than this. The dilution of this transfer plasma followed by a co-infusion with the perfusion puffer through the side arm of a langendorff system is one applicable option for transfer experiments.
38. Obtain the required blood (in our study human blood was used) [9], anticoagulated with heparin [28]. Centrifugate at 800 × *g* for 10 min (4°C), this avoids hemolysis.
39. Separate plasma from the blood cell compartment.
40. Add 2.5 ml of plasma to a centrifugal ultrafiltration unit (NMWCO 20kDa) and then insert the floater. Let tube with inserted floater sit for 5 min to allow filter to be completely wet.
41. Centrifugate at 2000–2500 × *g* for 35–40 min (4°C). Quickly obtain the filtrate (approximately 500 µl from 2.5 ml starting volume) from the floater and transfer it to clean tubes on ice.
42. Incubate equal amounts of filtrate (transfer plasma) and Krebs-Henseleit buffer (see MATERIAL) to prepare a stock solution for heart perfusion.

### Transfer experiments using cross-species transfer of donor plasma (e.g., human plasma) to isolated recipient mouse hearts in a langendorff apparatus • TIMING 102–105 min

43. Have Krebs-Henseleit solution (see MATERIAL) at hand, which should be prepared daily.
44. Turn on water baths, heat to 39°C (final temperature in perfusion chamber will be approximately 37°C due to heat loss *via* tubing).
45. Gas Krebs-Henseleit with carbogen (95%O_2_, 5%CO_2_) for 15 min to obtain a pH of 7.4 in the perfusate. Have Krebs-Henseleit cycle through the apparatus and ensure that perfusion chamber is filled with perfusate.
46. Set up a precise medical perfusor. From the perfusor, a line will run to the side arm of the perfusion chamber. Fill this solution in the perfusor syringe and attach it to the langendorff apparatus. This way, liquids such as transfer plasma can be co-infused to the isolated hearts.
47. Go ahead with Steps 27–30. Attach the cannulated heart to the langendorff apparatus (**Fig. 7D**).
48. During this procedure, the perfusor should be in running mode.
49. Set perfusion pressure to 80–100 mmHg (constant pressure mode).
50. Attach pacemaker electrode and stimulate the heart at a constant rate of 600 bpm.
51. Start the recording software to record at the minimum, aortic pressure and coronary flow.
52. Allow equilibration of the heart (20 min). The net coronary flow will hereafter equilibrate to 2.5–3.5 ml/min. Maintenance of an adequate temperature (37°C–38°C is required. If possible the chamber temperature should therefore be monitored.
53. Start the co-infusion of transfer plasma by infusing 1 ml/min when coronary equilibrium flow is 2.5 ml/min. Adapt the flow by increasing perfusor speed accordingly to maintain this relation. Continue for 20 min.
**CRTICAL STEP**: This requires a higher amount of blood and resulting transfer plasma and should be considered when planning the experiment. Off note, when investigating the contribution of the NO system to conditioning [9,11,29,30], all handling materials and liquids must be tested for respective nitrite contaminations using highly sensitive techniques [31].
54. Switch off the perfusion line including transfer plasma perfusor to establish global ischemia. Continue global ischemia for 30 min.
55. Reperfuse the heart for 60 min, and then detach the heart from the cannula and process as required.

**Table 2.** Troubleshooting.

Step	Problems	Causes	Suggestions
5,7	Sedation depth insufficient	Insufficient drug concentration for the individual	Have the mouse breath isoflurane through mask until depth of narcosis is sufficient.
7	Intubation failure	Difficulties locating larynx	Place cold light strictly in front of the larynx, perform second attempt or have a second operator perform intubation. If mouse is without ventilation and spontaneous breath for more than 1 min, consider killing the mouse.
7	Ventilation failure	Rupture of the lung, tension pneumothorax	Kill the mouse, check ventilation system, *e.g.*, using an artificial lung, decrease tidal volume and ventilation pressures.
18–20	Bleeding into pericardium	Surgical manipulation on fragile structures	Kill the mouse.
20	Difficulties locating coronary artery	The coronary anatomy varies in strains	Gently push the heart out from the thorax by pushing against the right sight of the thorax. Slightly increase thoracotomy length to the right sight while avoiding pneumothoraces. In some strains, *e.g.*, lacking endothelial NO synthase, visual location of the coronary artery is particularly difficult. In these cases, the typical location of the coronary could be assumed and the ligation performed. Ligation success will be obtained after staining for AAR and infarction-to-risk area.
28,31	Large remaining amount of blood in the excised heart	Insufficient anticoagulation	Add heparin (*e.g.*, 5000–10000 IE per 10 ml preparation buffer) to the buffer in the cooling plate.
31	Difficulties in aortic cannulation	Short aorta	Aorta should be at a sufficient length. Do no resect below the aortic valve.
31	Leakage from the cannulated aorta	Insufficient ligation	Apply a second ligation quickly. Bear in mind that the total preparation time should stay limited to avoid further global hypoxia/ischemia. Omit the heart if leakage cannot be located.
32	Coronary ligation cannot be re-located	Specific anatomical characteristics in particular mouse strain	Leave the primary suture unligated in the thorax during *in vivo* reperfusion period. After heart excision re-ligates this suture.
52	Arrhythmia	Incorrect composition of perfusion buffer or long heart preparation time	Check pacemaker set-up and buffer composition. Keep track of your preparation time (until cannulation). Try to further minimize preparation time.

## ANTICIPATED RESULTS

Following our protocol, we believe that investigators will be able to independently perform mouse intubation and anesthesia. Researchers will be able to reproducibly apply conditioning techniques and conduct infarction studies themselves. While the aspect of myocardial I/R naturally applies particularly to investigators in the field of cardiovascular research, conditioning and particularly rIPC can be used by many researches in different fields, *e.g.*, acute medical care, neurology (rIPC in the setting of stroke [32]), nephrology or hepatology (kidney and liver transplantation as clinically relevant models of organ I/R). Arguably, this is a model for use in mice lacking many aspects of cardiovascular patients. However, it is presumably the best approach to detect novel signaling components of myocardial I/R injury warranted to reduce mortality in patients. At all steps of the concurrent protocol we have put particular emphasis on the standardization and reproducibility. This pertains to our simplified conditioning technique as well as myocardial I/R injury, MRI and finally the analysis of tissues.

### Infarct sizes

Complete I/R injury will be best analyzed after 24 h by TTC (exemplary micrographs in **Fig. S2** and for imaging in **Fig. S3**). Less reperfusion will result in small infarct-to-risk sizes. Longer reperfusion will cause remodeling to an extend that a final scar is formed. Our *in vivo* I/R (30 min ischemia, 24 h reperfusion) model typically produces a medium infarct size of approximately 35% (infarct per AAR) with a standard deviation of 4%–5% as determined by TTC staining. Slight yet insignificant differences may occur between investigators. We therefore advice that one investigator should perform at least the critical coronary ligation step per study.

### Ischemic conditioning

Ischemic conditioning techniques are probably the best available cardioprotective methods. rIPC reduces infarction-to-risk by approximately 50% in both C57BL/6 and NMRI wild-type mice [9]. The latter have slightly thicker legs. Application of an IPC regimen (4 × 5 min coronary occlusion followed by 5 min of reperfusion) produces an even more pronounced decrease in infarction size. AAR as determinant of reproducibility did not differ. Based on an additional study in mice, we achieved a mean infarct size of 36% ± 5% (mean ± SD) of the area risk with a reduction in I/R injury to 18% ± 6% after the described rIPC maneuver.

Taking together, the infarction model has been applied in a wide variety of studies and has proven excellent reproducibility [9,16,24,33,34]. The conditioning techniques represent a simple yet highly effective tool to reduce lethal myocardial I/R injury. In combination, this bioapproach is a valuable animal model for translational research in the scope of myocardial infarction and cardioprotection.

**Table 3.** Troubleshooting LDPI.

Problem	Possible reason	Solution
Poor LDPI signal quality	Insufficient skin area for adequate measurements	• Gently remove hindlimb hair, *e.g.*, by using depilatory creme • Avoid shaving as excessive mechanical manipulation can significantly alter skin perfusion
Changing LDPI signal	Changes in light conditions	• Try to maintain the same light conditions throughout the experiments and adjust the threshold to the background light conditions • Maintain distance of the detector to the hindlimb at the exact same position (typically 20 cm)
Changing LDPI signal	Poor tissue perfusion due to changes in ambient temperature	• Maintain the exact same temperature throughout the experiment particularly avoiding cooling draughts • A closed room for experiments has been proven to be superior for LDPI experimental outcomes
No recovery of perfusion signal	• Cuff too small • Hindlimb has moved	• Change to larger cuffs • Fix hindlimb properly

## TROUBLESHOOTING

**Table 1** lists potential (dis-)advantages of the different *in vivo* models. Troubleshooting advice can be found in **Table 2** and **Table 3**.

## Abbreviation used

AAR: area at risk
ECG: electrocardiogram
i.p.: intraperitoneally
I/R: ischemia/reperfusion
LCA: left coronary artery
LGE: late-gadolinium enhanced
LV: left ventricle
LDPI: laser Doppler perfusion imaging
MRI: magnetic resonance imaging
(r)IPC: (remote) ischemic preconditioning
TTC: 2,3,5-triphenyl-2H-tetrazolium chloride
US: Ultrasound

## Supplementary information

**Figure S1**. Schematic outline of anesthesia induction and maintenance.

**Figure S2**. Exemplary micrographs after I/R.

**Figure S3**. Original ultrasound registration.
